# Phlegmonous Gastritis in a Bariatric Patient After Sleeve Gastrectomy

**DOI:** 10.7759/cureus.5898

**Published:** 2019-10-12

**Authors:** Saqib Saeed, Sara Alothman, Kashif Saeed, Leaque Ahmed, Sanjiv Gray

**Affiliations:** 1 Surgery, Harlem Hospital Center, New York, USA; 2 Surgery, University of Central Florida College of Medicine, Orlando, USA

**Keywords:** sleeve gastrectomy, phlegmonous gastritis, bariatric

## Abstract

Phlegmonous gastritis is a rare and progressive fatal condition that affects the mucosa and submucosa of the gastric wall. It can be localized or diffuse, affecting the entire stomach. It usually presents with upper gastrointestinal symptoms, such as nausea, vomiting, and hematemesis, along with systemic symptoms, including fever, chills, and fatigue. Risk factors include mucosal injury, surgery, hypoacidity, and immunosuppression that can be seen in human immunodeficiency virus (HIV)-positive or alcoholic patients. We present a case of phlegmonous gastritis which developed after a laparoscopic sleeve gastrectomy. The patient presented with epigastric pain, nausea, and chills two months post-sleeve gastrectomy. The diagnosis was made with computed tomography (CT) scan of the abdomen. She was managed successfully with CT-guided drainage and antibiotics.

## Introduction

Phlegmonous or suppurative gastritis (PG) is a rare, life-threatening bacterial infection of the gastric wall affecting the submucosa and muscularis propria first described by Cruveilhier in 1862 [1]. Infection can be localized in 5% - 15% while being diffuse and phlegmonous in other cases [2]. The Streptococcus species is the most common pathogen related to PG, accounting for 68% - 75% of all cases [3-4]. The pathophysiology of PG is not fully understood. Patients present with abdominal pain, nausea, vomiting, and fever. Risk factors include alcoholism, increased age, malnutrition with a low albumin level, low socioeconomic status, immunocompromised patients, and uncontrolled diabetes mellitus [4]. There is no consensus on the proper management of the disease. In this study, we present a case of acute phlegmonous gastritis following a sleeve gastrectomy which was successfully managed by computed tomography (CT)-guided drainage.

## Case presentation

**Figure 1:**
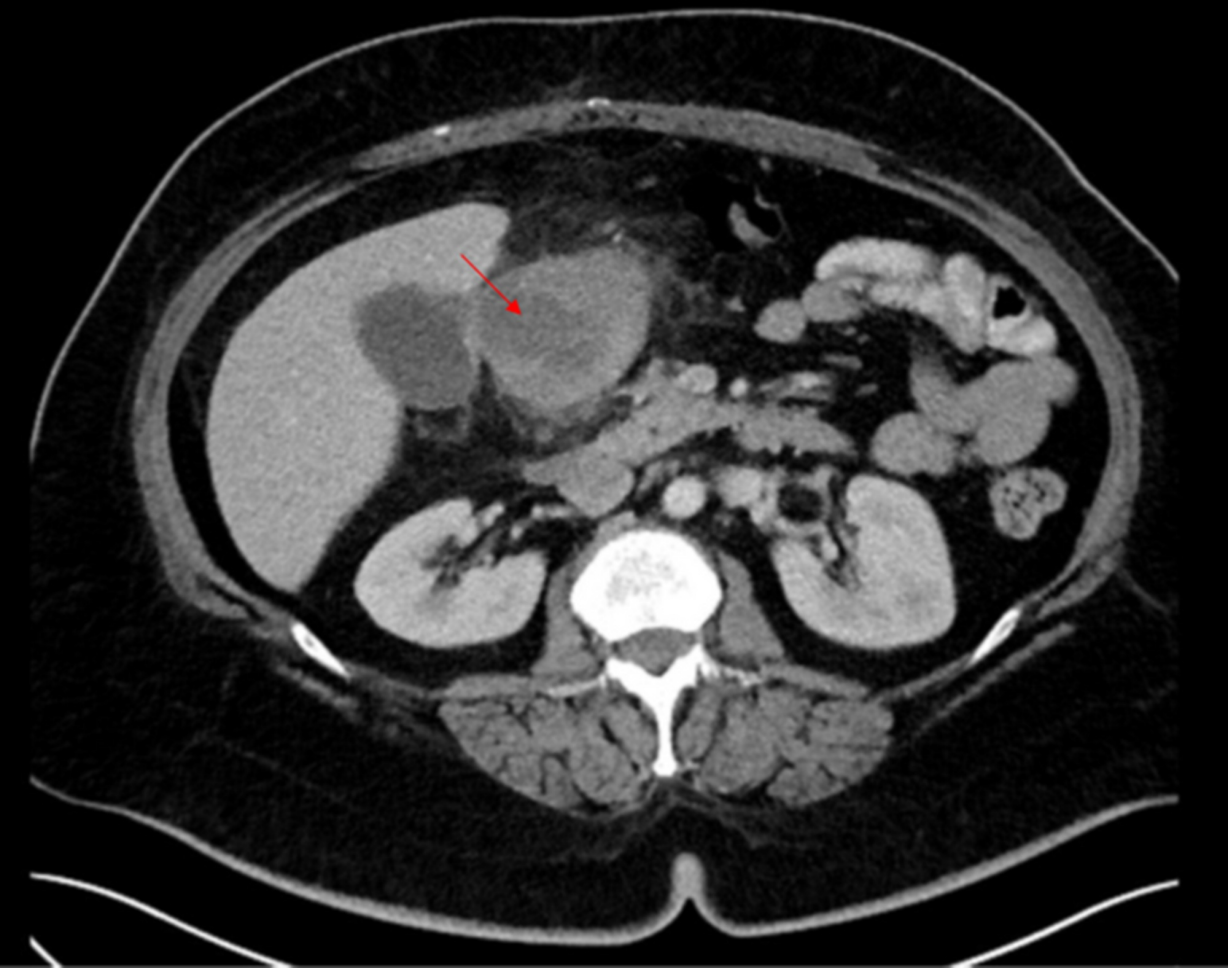
Computed tomography scan of the abdomen/pelvis showing phlegmonous gastritis

A 59-year-old female underwent laparoscopic sleeve gastrectomy for morbid obesity. The procedure was uneventful and she was discharged on postoperative Day 2. The patient was seen in the clinic one and two weeks after surgery. She was doing well at that time. On her two-month postop follow-up visit, she was referred to the emergency room for a four-day history of epigastric pain, nausea, fatigue, dizziness, and chills without subjective fever. On physical exam, she was noted to be diaphoretic. Her vital signs revealed a temperature of 97.5° F, blood pressure of 93/65, a pulse of 100, and a respiratory rate of 17 breaths/minute. She had diffuse abdominal tenderness without rebound or guarding. Laboratory values revealed a white cell count of 15.6 (K/uL) with 82.6% neutrophils, hemoglobin - 10.8 (g/dl), hematocrit - 33.9%, platelets - 187 K/uL, sodium - 143 mmol/l, potassium - 4.00 mmol/l, chloride - 104 mmol/l, carbon dioxide - 28 mmol/l, blood urea nitrogen - 18 mg/dl, and creatinine - 0.9 mg/dl. CT scan of the abdomen and pelvis showed marked mucosal thickening within the antrum and pyloric region of the stomach, along with a hypodense fluid collection within the stomach wall with a mean density of 24 Hounsfield units. There was associated adjacent fat stranding. This constellation of findings were suggestive of gastritis with possible abscess formation (Figure 1).

**Figure 2:**
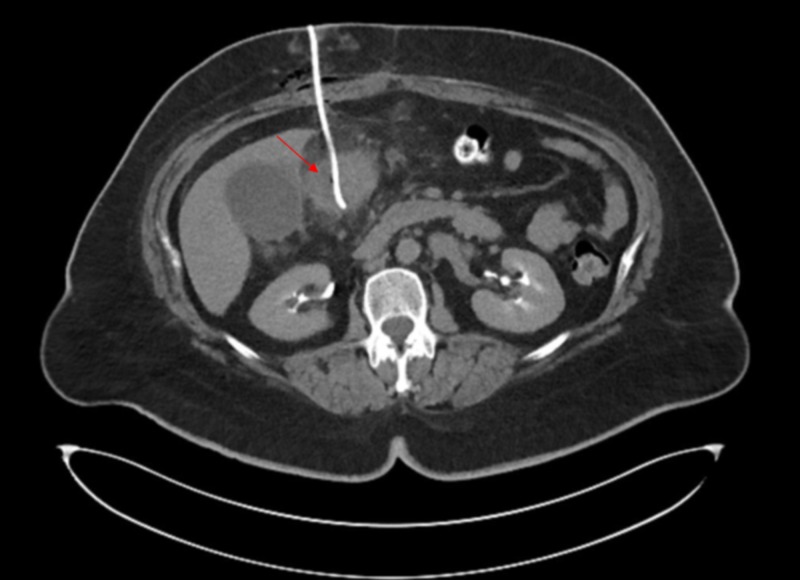
Computed tomography scan showing the pigtail catheter in place

The patient was started on vancomycin, 1 gram intravenous every 12 hours, and Zosyn®, 3.375 g intravenous every eight hours (Wyeth Pharmaceuticals LLC, Philadelphia, PA). She underwent CT-guided drainage with an 8-French locking pigtail catheter placed into the collection which was then attached to a Jackson-Pratt® bulb suction (Cardinal Health™, Waukegan, IL) (Figure 2).

A total of 10 mL of thick purulent fluid was aspirated and delivered to the laboratory for further evaluation. A completion CT scan demonstrated the drain to be within the collection with minimal remaining fluid within the abscess and no evidence of hematoma, peritoneal fluid leakage, or other complication. Gram stain revealed moderate gram-positive cocci in pairs and in clusters. The culture grew predominantly Streptococcus sanguinis and few coagulase-negative Staphylococci. The Streptococcus sanguinis was susceptible to penicillin and vancomycin. The patient was discharged on amoxicillin, 875 mg, and clavulanate, 125 mg every 12 hours, for seven days. She was seen in the clinic four days after the drainage and was doing well with no complaints. The pigtail catheter was removed. The patient continued to follow-up in the bariatric clinic as per our center protocol. She underwent a repeat CT of the abdomen one month after the drainage which showed a complete resolution of the hypoattenuating fluid collection within the antrum and a significant reduction in the adjacent fat stranding visualized on the prior study.

## Discussion

Phlegmonous gastritis (PG) is a rare clinical entity that can occur after gastric surgery (in our case, four months after a laparoscopic sleeve gastrectomy). It can be fatal if not diagnosed promptly. The risk factors include advanced age, mucosal injury, hypoacidity, immunosuppression, prior gastric surgery, or biopsy. It presents with epigastric pain and vomiting. Other symptoms include fevers, chills, diarrhea, and hematemesis [4].

The diagnosis can be made using abdominal ultrasound, CT scan, endoscopy, and endoscopic ultrasound. Our patient’s CT findings showed pre-pyloric wall thickening with an intramural hypodense area with rim enhancement which represented an abscess.

The exact pathogenesis is unknown [3-4]. On histopathology, there is acute inflammation of the gastric submucosa. The bacteriology of the abscess includes Streptococcus, which is isolated 70% of the time, as well as the Staphylococcus species, Escherichia coli, hemophilic influenza, Proteus, and Clostridium. In our case, the culture grew Streptococcus sanguinis which is concomitant with the most common organism isolated in the literature.

The disease is rapidly fatal if early treatment is not initiated. The mortality ranges from 27% in modern series and as high as 92% in earlier series [6]. Treatment is controversial. General principles of early goal-directed therapy with fluid resuscitation, timely antibiotic administration, and control of the infection are appropriate [5]. Our patient was successfully treated with antimicrobials and image-guided drainage.

## Conclusions

Even though laparoscopic sleeve gastrectomy is considered to be one of the safest surgical procedures, lethal complications can still occur. High clinical suspicion with early recognition and prompt treatment is warranted. The patient can be managed successfully with antibiotics and image-guided drainage. Minimal invasive treatment and antibiotics can result in good outcomes. In our case, the outcome was favorable. Surgery should be done for refractory cases to prevent the need for gastrectomy and death.
